# Exploring adverse events associated with vosoritide monotherapy: Insights from the FDA Adverse Event Reporting System

**DOI:** 10.1371/journal.pone.0341323

**Published:** 2026-01-29

**Authors:** Xiaomin Li, Xiangli Luo, Fei Liu, Chaolu Wang, Xiaoming Li, Huaixi Yu

**Affiliations:** 1 Department of Orthopedics, Wangjing Hospital, China Academy of Chinese Medical Sciences, Beijing, People’s Republic of China; 2 Department of Orthopedics, Gansu Provincial People’s Hospital, Lanzhou, People’s Republic of China; 3 Department of Oral Implantology, The Affiliated Stomatological Hospital of Xuzhou Medical University, Xuzhou, People’s Republic of China; 4 Department of Orthopedics, The Affiliated Huai’an Hospital of Xuzhou Medical University, Huaian, People’s Republic of China; The University of Hong Kong, CHINA

## Abstract

**Background:**

Dwarfism, a condition characterized by short stature, has been the focus of therapeutic advancements with the emergence of novel peptide drugs. Vosoritide, indicated for certain types of dwarfism, has shown therapeutic potential in clinical trials. However, a comprehensive safety profile is essential for its clinical application. The current literature lacks a detailed assessment of vosoritide’s safety, indicating a significant gap that this study aims to address.

**Methods:**

We conducted a retrospective pharmacovigilance study by analyzing the FDA Adverse Event Reporting System (FAERS) database to evaluate adverse events (AEs) associated with vosoritide monotherapy. The study employed a case/non-case methodology and applied signal detection algorithms, including the Reporting Odds Ratio (ROR), Proportional Reporting Ratio (PRR), Bayesian Confidence Propagation Neural Network (BCPNN), and Multi-Item Gamma Poisson Shrinker (MGPS), to identify AE signals related to vosoritide use.

**Results:**

The study included 9319269 reports from the FAERS database, covering the period from 2022 to 2023, with 274 reports specifically citing vosoritide. A significant number of AEs were identified, with a notable incidence in the pediatric population. The most frequently reported AEs were related to endocrine disorders, including altered growth hormone levels and glucose homeostasis issues. Other affected system organ classes (SOCs) were infections and infestations, as well as skin and subcutaneous tissue disorders. Specific preferred terms (PTs) associated with vosoritide included “growth deceleration,” “glycemic dysregulation,” and “local injection site reactions.”

**Conclusion:**

The findings from this study underscore the importance of close monitoring of vosoritide treatment, particularly in pediatric patients. The identification of both expected and unexpected AEs highlights the necessity for ongoing pharmacovigilance and further research to fully understand the safety profile of vosoritide in clinical practice. This study contributes to the broader field by emphasizing the critical need for patient safety considerations in the development of new therapeutic agents.

## Introduction

Vosoritide as a therapeutic intervention, has marked a significant milestone in the management of achondroplasia, the most common form of short-limbed dwarfism [1]. As a C-type natriuretic peptide, vosoritide holds promise in not only enhancing linear growth but also potentially ameliorating the broader spectrum of achondroplasia-related complications [2]. However, with any novel pharmaceutical agent, the elucidation of its safety profile in diverse patient populations is paramount [3]. Clinical trials provide the foundational evidence for the efficacy and safety of new drugs, yet they may not fully capture the breadth of AEs that may emerge in real-world scenarios [4]. The heterogeneity of patient responses outside controlled environments, coupled with the unique challenges of long-term drug exposure, necessitates ongoing pharmacovigilance [5]. In this context, the FDA Adverse Event Reporting System (FAERS) emerges as a critical tool, offering a repository of spontaneously reported AEs that can inform post-marketing surveillance [6]. This study leverages the FAERS database to conduct a retrospective pharmacovigilance analysis of vosoritide monotherapy, aiming to identify, quantify, and evaluate AEs associated with its use. By employing advanced signal detection algorithms, including the Reporting Odds Ratio (ROR), Proportional Reporting Ratio (PRR), Bayesian Confidence Propagation Neural Network (BCPNN), and Multi-Item Gamma Poisson Shrinker (MGPS), we aim to uncover potential safety signals that may have eluded detection in pre-approval studies [7]. The importance of this research is underscored by the need to protect vulnerable patient populations, such as children with achondroplasia, who may be more susceptible to AEs due to their developmental stage and unique physiology [8]. Furthermore, understanding the safety profile of vosoritide is not only crucial for its current use but also for the development of future therapeutics for achondroplasia and similar conditions [9]. This study’s findings will contribute to the existing body of literature by providing insights into the real-world safety of vosoritide. It will also address gaps in knowledge regarding the long-term tolerability and safety of this drug in pediatric populations [10]. By doing so, this research will aid healthcare providers in making informed decisions regarding the risk-benefit ratio of vosoritide therapy [11]. The implications of this study extend beyond the immediate safety assessment of vosoritide. It will also contribute to the broader field of pharmacovigilance, highlighting the importance of continuous monitoring in the post-marketing phase of drug development [12]. Moreover, the study will provide a template for evaluating the safety profiles of novel drugs targeting rare genetic conditions [13]. In conclusion, this pharmacovigilance study aims to provide a comprehensive evaluation of the AEs associated with vosoritide monotherapy, contributing to the ongoing discourse on its safety and efficacy in the treatment of achondroplasia. The findings will be instrumental for healthcare providers, regulatory authorities, and patients in making informed decisions regarding the use of vosoritide.

## Methods

### Data acquisition

**Fig 1:**
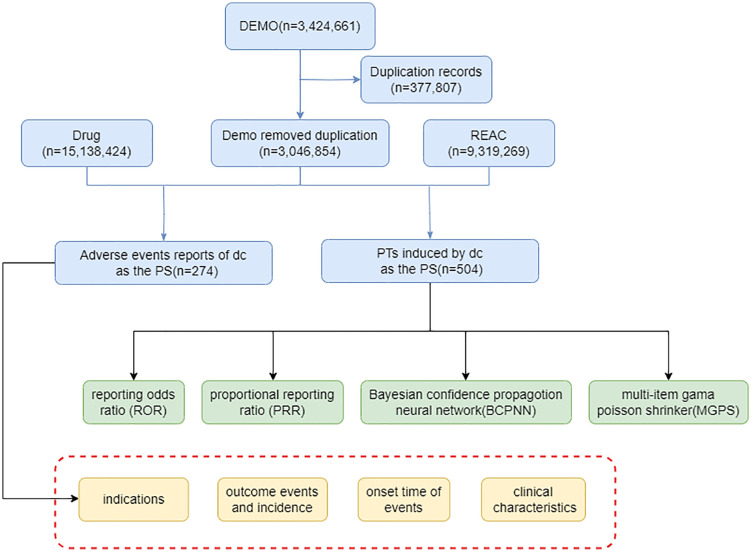
The flow diagram of selecting Vosoritide Monotherapy -related AEs from FAES database.

Our pharmacovigilance study was based on data extracted from the FDA Adverse Event Reporting System (FAERS), covering the period from January 2022 to December 2023. The FAERS database provided a comprehensive collection of adverse event reports associated with drug usage. We focused on extracting data related to vosoritide, ensuring that the information was relevant to our study’s objectives (Fig 1).

### Inclusion and exclusion criteria

The inclusion criteria for our analysis were defined to select reports that explicitly mentioned vosoritide as the suspect medication. We excluded reports that did not specify vosoritide or where the relationship between the drug and the reported adverse event was unclear. We also excluded any duplicate reports to ensure the accuracy and reliability of our findings.

### Data processing

The raw data obtained from FAERS underwent a thorough cleaning process to standardize terminologies and remove any inconsistencies. This step was crucial for enhancing the quality of our analysis and ensuring that the data was suitable for pharmacovigilance signal detection.

### Signal detection algorithms

We employed several disproportionality analysis methods to identify potential safety signals associated with vosoritide (Table 1).

**Table 1:** The specific formulas for the four algorithms.

Method	Formula	Threshold
ROR	$\text{ROR}=\frac{\text{a}\;/\;\text{c}}{b\;/\;d}$	a ≥ 3 ROR ≥ 2 95%CI (lower limit) > 1
	$SE(lnROR)=\sqrt{\frac{\text{1}}{\text{a}}+\frac{\text{1}}{\text{b}}+\frac{\text{1}}{\text{c}}+\frac{\text{1}}{\text{d}}}$	
	$\text{9}\text{5}\%CI=\;{\text{e}}^{\text{ln}(ROR)\pm \text{1}.\text{9}\text{6}se}$	
PRR	$\text{PRR}=\frac{\text{a}\;/\;(\text{a}+\text{b})}{c\;/\;(c+d)}$	a ≥ 3 PRR ≥ 2 95%CI (lower limit) > 1
	$SE(lnPRR)=\sqrt{\frac{\text{1}}{\text{a}}-\frac{\text{1}}{\text{a}+\text{b}}+\frac{\text{1}}{\text{c}}-\frac{\text{1}}{\text{c}+\text{d}}}$	
	$\text{9}\text{5}\%CI=\;{\text{e}}^{\text{ln}(PRR)\pm \text{1}.\text{9}\text{6}se}$	
BCPNN	$\text{IC}={\text{log}}_{\text{2}}\frac{p(x,\;y)}{p(x)p(y)}=\;{log}_{\text{2}}\frac{a(a+b+c+d)}{\left(a+b)(a+c\right)}$	IC025 > 0
	$\text{E}(\text{IC})={\text{log}}_{\text{2}}\frac{\left(a+\gamma_{\text{1}\text{1}})(a+b+c+d+\alpha )(a+b+c+d+\beta \right)}{\left(a+b+c+d+\gamma )(a+b+\alpha_{\text{1}})(a+c+\beta_{\text{1}}\right)}$	
	$\text{V}(\text{IC})=\frac{\text{1}}{{\left({ln}_{\text{2}}\right)}^{\text{2}}}[\frac{(a+b+c+d)-a+\gamma -\gamma_{\text{1}\text{1}}}{\left(a+\gamma_{\text{1}\text{1}}\right)(\text{1}+a+b+c+d+\gamma )}+\frac{(a+b+c+d)-(a+b)+a-\alpha_{\text{1}}}{\left(a+b+\alpha_{\text{1}}\right)(\text{1}+a+b+c+d+\alpha )}+\frac{(a+b+c+d+\alpha )-(a+c)+\beta -\beta_{\text{1}}}{\left(a+b+\beta_{\text{1}}\right)(\text{1}+a+b+c+d+\beta )}]$	
	$\gamma =\gamma \text{11}\frac{\left(a+b+c+d+\alpha )(a+b+c+d+\beta \right)}{\left(a+b+\alpha_{\text{1}}\right)(a+c+\beta_{\text{1}})}$	
	$\text{IC}-\text{2SD}=\text{E}(\text{IC})-\text{2}\;\sqrt{\text{V}(\text{IC})}$	
EBGM	$\text{EBGM}=\frac{a(a+b+c+d)}{\left(a+c)(a+b\right)}$	EBGM05 > 2
	$SE(lnEBGM)=\sqrt{\frac{\text{1}}{\text{a}}+\frac{\text{1}}{\text{b}}+\frac{\text{1}}{\text{c}}+\frac{\text{1}}{\text{d}}}$	
	$\text{9}\text{5}\%CI=\;{\text{e}}^{\text{ln}(EBGM)\pm \text{1}.\text{9}\text{6}se}$	

**Note:** a: Number of reports with both exposure to the target drug (vosoritide) and occurrence of the target adverse event (AE); b: Number of reports with exposure to the target drug but no occurrence of the target AE; c: Number of reports without exposure to the target drug but with occurrence of the target AE; d: Number of reports without exposure to the target drug and no occurrence of the target AE; x: Exposure status of the target drug (x = 1 indicates exposure, x = 0 indicates non-exposure); y: Occurrence status of the target AE (y = 1 indicates occurrence, y = 0 indicates non-occurrence); p(x,y): Joint probability of simultaneous occurrence of drug exposure and AE; p(x): Marginal probability of drug exposure; p(y): Marginal probability of AE occurrence; γ11: Prior count of simultaneous presence of drug exposure and AE in the BCPNN model; α: Prior parameter related to drug exposure in the BCPNN model; β: Prior parameter related to AE occurrence in the BCPNN model; γ: Global prior parameter in the BCPNN model (γ = γ11×(a + b + c + d + α)(a + b + c + d + β)/[(a + b + α1)(a + c + β1)]); α1: Prior count of drug exposure in the BCPNN model; β1: Prior count of AE occurrence in the BCPNN model; E(IC): Expected value of the Information Component (IC) in the BCPNN model; V(IC): Variance of the Information Component (IC) in the BCPNN model; IC025: 2.5th percentile of the Information Component (IC) in the BCPNN model; EBGM05: 5th percentile of the Empirical Bayes Geometric Mean (EBGM) in the MGPS model; se: Standard error of the corresponding statistic. ROR = Reporting Odds Ratio; PRR = Proportional Reporting Ratio; BCPNN = Bayesian Confidence Propagation Neural Network; IC = Information Component; EBGM = Empirical Bayes Geometric Mean; CI = Confidence Interval; SE = Standard Error; a = Number of reports with both exposure to vosoritide and occurrence of the target adverse event (AE); b = Number of reports with exposure to vosoritide but no occurrence of the target AE; c = Number of reports without exposure to vosoritide but with occurrence of the target AE; d = Number of reports without exposure to vosoritide and no occurrence of the target AE; γ11 = Prior count of simultaneous presence of drug exposure and AE in the BCPNN model; α = Prior parameter related to drug exposure in the BCPNN model; β = Prior parameter related to AE occurrence in the BCPNN model; γ = Global prior parameter in the BCPNN model; α1 = Prior count of drug exposure in the BCPNN model; β1 = Prior count of AE occurrence in the BCPNN model; E(IC) = Expected value of IC; V(IC) = Variance of IC; IC025 = 2.5th percentile of IC; EBGM05 = 5th percentile of EBGM; se = Standard error.

### Statistical analysis

The statistical analysis was performed using the R software, version 4.1. The data was analyzed to calculate the ROR, PRR, and to generate the BCPNN and MGPS scores. We considered a signal to be significant if the ROR or PRR was greater than 2, the BCPNN Information Component (IC) was greater than 1, and the MGPS Empirical Bayes Geometric Mean (EBGM) was greater than 2.

## Result

### General characteristics of the study population

The analysis of the FAERS database yielded a total of 266 reports associated with vosoritide use from 2022 to 2023. The majority of these reports were submitted in 2023, indicating a recent increase in the monitoring of vosoritide’s safety profile. The gender distribution was predominantly male, aligning with the typical patient demographic for achondroplasia treatment. However, a significant portion of reports did not specify the sex, highlighting a data capture limitation. Notably, all weight categories had minimal or no reports, suggesting potential underreporting or a focus on pediatric populations where weight may not be a primary reporting criterion. A substantial majority of the reports originated from consumers, emphasizing the role of patient advocacy and engagement in safety monitoring. Reports from healthcare professionals were minimal, indicating a potential area for enhanced professional reporting. Geographically, the United States contributed the most reports, suggesting a higher utilization rate or a more robust reporting infrastructure. The majority of reports indicated subcutaneous administration, which is the standard route for vosoritide administration. In terms of outcomes, a notable proportion of reports described serious outcomes, including hospitalizations and life-threatening conditions, underscoring the critical nature of safety surveillance. The time to onset (TTO) varied, with a significant number of events occurring beyond 60 days, indicating a need for long-term monitoring post-administration (Table 2).

**Table 2:** Characteristics of reports associated with vosoritide.

Variable	Total
**Year**	
2022	129 (47.08)
2023	145 (52.92)
**Sex**	
Female	17 (6.20)
Male	16 (5.84)
Unknown	241 (87.96)
**Age**	
<18	23 (8.39)
Unknown	251 (91.61)
**Weight**	
<60 kg	8 (2.92)
60 ~ 80 kg	0 (0.00)
>80 kg	0 (0.00)
Unknown	266 (97.08)
**Reporter**	
Consumer	268 (97.81)
Physician	3 (1.09)
Unknown	2 (0.73)
Pharmacist	1 (0.36)
**Reported countries**	
United States	232 (84.67)
Other	42 (15.33)
**Route**	
Subcutaneous	216 (78.83)
Other	58 (21.17)
**Outcomes**	
Other serious	35 (55.56)
Hospitalization	20 (31.75)
Life threatening	3 (4.76)
Disability	2 (3.17)
Congenital anomaly	1 (1.59)
Death	1 (1.59)
Required intervention	1 (1.59)
**TTO**	
=<6 days	22 (17.74)
7 ~ 28 days	10 (8.06)
29 ~ 59 days	13 (10.48)
>=60 days	23 (18.55)
Unknown	56 (45.16)

**Note:** TTO = Time to Onset.

### Analysis of System Organ Class (SOC) for vosoritide-related adverse events

The pharmacovigilance analysis of FAERS data for vosoritide revealed a range of adverse events across different System Organ Classes (SOCs). Notably, “Congenital, Familial, and Genetic Disorders” emerged with a significant reporting odds ratio (ROR) of 6.84, suggesting a pronounced signal for this class of disorders. The proportional reporting ratio (PRR) was also elevated at 6.74, with a chi-square value of 44.07, reinforcing the observed association. “General Disorders and Administration Site Conditions” were reported frequently, with a total of 216 cases. The ROR and PRR for this category were 3.24 and 2.28, respectively, indicating a substantial signal for adverse events in this area. The information component (IC) and empirical Bayes geometric mean (EBGM) values were 1.19 and 2.28, respectively, further supporting the significance of these findings.

“Ear and Labyrinth Disorders” also showed a notable signal, with an ROR and PRR of 2.84 and 2.82, respectively. The chi-square value was 7.09, and the IC was 1.50, indicating a potential area of concern. Other SOCs, such as “Infections and Infestations,” “Respiratory, Thoracic and Mediastinal Disorders,” and “Skin and Subcutaneous Tissue Disorders,” exhibited moderate signals with RORs ranging from 1.49 to 1.27. These findings suggest a need for further investigation to understand the nature of these associations. Interestingly, “Psychiatric Disorders” and “Investigations” showed lower RORs and PRRs, with values below 1, indicating a less pronounced association with vosoritide use. The negative IC values for these categories suggest a lower likelihood of these adverse events being related to the drug. The analysis underscores the importance of ongoing monitoring for adverse events in the “Congenital, Familial, and Genetic Disorders” category, given the strong signals detected. It also highlights the need for vigilant surveillance in other reported SOCs to ensure patient safety and to inform clinical decision-making (Table 3).

**Table 3:** SOC of vosoritide -related AEs from FAES database.

SOC	Case Reports	ROR (95% CI)	PRR (95% CI)	chisq	IC (IC025)	EBGM (EBGM05)
Congenital, familial and genetic disorders^*^	9	6.84 (3.54, 13.23)	6.74 (3.53, 12.87)	44.07	2.75 (1.85)	6.74 (3.88)
General disorders and administration site conditions^*^	216	3.24 (2.71, 3.86)	2.28 (2.07, 2.51)	190.83	1.19 (0.96)	2.28 (1.97)
Ear and labyrinth disorders^*^	6	2.84 (1.27, 6.36)	2.82 (1.26, 6.3)	7.09	1.5 (0.42)	2.82 (1.44)
Infections and infestations^*^	46	1.49 (1.1, 2.02)	1.45 (1.1, 1.91)	6.82	0.53 (0.1)	1.45 (1.12)
Respiratory, thoracic and mediastinal disorders^*^	34	1.48 (1.05, 2.1)	1.45 (1.04, 2.02)	4.98	0.54 (0.04)	1.45 (1.08)
Skin and subcutaneous tissue disorders	33	1.27 (0.9, 1.81)	1.26 (0.9, 1.76)	1.83	0.33 (−0.17)	1.26 (0.94)
Vascular disorders	12	1.25 (0.7, 2.21)	1.24 (0.7, 2.19)	0.57	0.31 (−0.48)	1.24 (0.77)
Nervous system disorders	45	1.22 (0.9, 1.66)	1.2 (0.91, 1.58)	1.65	0.27 (−0.17)	1.2 (0.93)
Musculoskeletal and connective tissue disorders	24	0.86 (0.57, 1.29)	0.87 (0.59, 1.29)	0.53	−0.21 (−0.79)	0.87 (0.61)
Gastrointestinal disorders	33	0.79 (0.56, 1.13)	0.81 (0.58, 1.13)	1.66	−0.31 (−0.81)	0.81 (0.6)
Metabolism and nutrition disorders	6	0.61 (0.27, 1.36)	0.61 (0.27, 1.36)	1.5	−0.71 (−1.78)	0.61 (0.31)
Psychiatric disorders	10	0.35 (0.18, 0.65)	0.36 (0.2, 0.66)	12.17	−1.48 (−2.34)	0.36 (0.21)
Investigations	7	0.21 (0.1, 0.45)	0.23 (0.11, 0.48)	19.82	−2.15 (−3.16)	0.23 (0.12)
Injury, poisoning and procedural complications	8	0.1 (0.05, 0.21)	0.12 (0.06, 0.24)	61.23	−3.09 (−4.04)	0.12 (0.07)

**Note:** SOC = System Organ Class; FAERS = FDA Adverse Event Reporting System; ROR = Reporting Odds Ratio; PRR = Proportional Reporting Ratio; CI = Confidence Interval; IC = Information Component (from BCPNN); EBGM = Empirical Bayes Geometric Mean (from MGPS); BCPNN = Bayesian Confidence Propagation Neural Network; MGPS = Multi-Item Gamma Poisson Shrinker; IC025 = 2.5th percentile of IC; EBGM05 = 5th percentile of EBGM. The asterisk (*) indicates indicators that exceed the threshold.

### Analysis of Preferred Terms (PT) for vosoritide-related adverse events

The detailed analysis at the Preferred Terms (PT) level for adverse events (AEs) related to vosoritide use uncovered a spectrum of reactions, predominantly focusing on injection site reactions and systemic effects. The most frequently reported AEs were injection site urticaria and injection site reaction, with considerable Reporting Odds Ratios (RORs) of 51.18 and 43.75, respectively. These high RORs, along with their corresponding Proportional Reporting Ratios (PRRs) and chi-square values, indicate a strong association between these events and vosoritide use. Other injection site-related AEs, such as erythema, swelling, and pain, also demonstrated significant signals, with RORs ranging from 35.54 to 28.19. These findings underscore the need for monitoring local tolerability when administering vosoritide. Systemic AEs like pyrexia and hair growth abnormal were noted, with RORs suggesting a potential link to vosoritide treatment. Notably, “hair growth abnormal” showed an exceptionally high ROR, pointing to a unique AE that may be specific to vosoritide’s mechanism of action or patient population. The analysis also identified AEs like kyphosis and knee deformity, which, despite being less frequent, exhibited very high RORs, indicating a potentially significant safety concern. These findings warrant further investigation to understand their clinical relevance and the underlying mechanisms. Overall, the PT-level analysis provides a nuanced view of the AEs associated with vosoritide, highlighting the importance of comprehensive monitoring and vigilant reporting to ensure patient safety. The data emphasizes the need for ongoing pharmacovigilance to capture both common and rare AEs in real-world settings (Table 4).

**Table 4:** Top 22 adverse event of vosoritide at the preferred terms level.

PT	Case Reports	ROR (95% CI)	PRR (95% CI)	chisq	IC (IC025)	EBGM (EBGM05)
Injection site urticaria^*^	10	51.18 (27.35, 95.81)	50.19 (27.34, 92.15)	480.97	5.65 (4.78)	50.06 (29.62)
Injection site reaction^*^	17	43.75 (26.96, 71)	42.31 (26.43, 67.72)	684.66	5.4 (4.72)	42.22 (28.15)
Injection site erythema^*^	26	38.03 (25.62, 56.45)	36.12 (24.89, 52.42)	887.26	5.17 (4.61)	36.05 (25.9)
Injection site swelling^*^	19	35.54 (22.46, 56.22)	34.23 (21.81, 53.73)	612.53	5.09 (4.45)	34.17 (23.28)
Injection site pain^*^	59	28.19 (21.49, 37)	25.01 (19.77, 31.64)	1364.54	4.64 (4.26)	24.98 (19.9)
Injection site rash^*^	5	21.16 (8.76, 51.08)	20.96 (8.68, 50.63)	94.97	4.39 (3.23)	20.94 (10.01)
Injection site pruritus^*^	6	13.82 (6.18, 30.91)	13.66 (6.12, 30.51)	70.43	3.77 (2.69)	13.65 (6.96)
Injection site bruising^*^	7	12.99 (6.16, 27.39)	12.82 (6.09, 27)	76.33	3.68 (2.67)	12.81 (6.86)
Pyrexia^*^	20	7.33 (4.69, 11.47)	7.08 (4.6, 10.9)	104.98	2.82 (2.19)	7.08 (4.87)
Hair growth abnormal^*^	4	92.26 (34.41, 247.37)	91.53 (34.35, 243.88)	356.44	6.51 (5.23)	91.09 (39.91)
Rash pruritic^*^	3	7.23 (2.32, 22.51)	7.2 (2.31, 22.44)	16.02	2.85 (1.43)	7.19 (2.78)
Urticaria^*^	6	5.03 (2.25, 11.25)	4.98 (2.23, 11.12)	19.13	2.32 (1.24)	4.98 (2.54)
Kyphosis^*^	4	384.27 (142.23, 1038.23)	381.23 (143.08, 1015.77)	1486.31	8.55 (7.26)	373.55 (162.62)
Knee deformity^*^	4	247.67 (91.99, 666.78)	245.71 (92.22, 654.68)	962.1	7.92 (6.64)	242.5 (105.88)
Ear infection^*^	6	22.08 (9.87, 49.4)	21.83 (9.77, 48.76)	119.15	4.45 (3.37)	21.8 (11.11)
Gastroenteritis viral^*^	3	19.44 (6.25, 60.53)	19.33 (6.2, 60.25)	52.12	4.27 (2.85)	19.31 (7.47)
Pallor^*^	3	10.1 (3.25, 31.45)	10.05 (3.22, 31.32)	24.45	3.33 (1.91)	10.05 (3.89)
Tonsillar hypertrophy^*^	3	221.43 (70.7, 693.52)	220.11 (70.62, 686.03)	646.68	7.77 (6.33)	217.54 (83.69)
Vomiting^*^	18	5.53 (3.46, 8.86)	5.37 (3.42, 8.43)	64.45	2.43 (1.76)	5.37 (3.62)
Deafness^*^	3	14.71 (4.72, 45.77)	14.62 (4.69, 45.57)	38.06	3.87 (2.45)	14.61 (5.65)
Syringomyelia^*^	5	3112.47 (1202.72, 8054.6)	3081.6 (1202.8, 7895.15)	13198.35	11.37 (10.11)	2641.52 (1192.16)

**Note:** PT = Preferred Term; ROR = Reporting Odds Ratio; PRR = Proportional Reporting Ratio; CI = Confidence Interval; IC = Information Component (from BCPNN); EBGM = Empirical Bayes Geometric Mean (from MGPS); BCPNN = Bayesian Confidence Propagation Neural Network; MGPS = Multi-Item Gamma Poisson Shrinker; IC025 = 2.5th percentile of IC; EBGM05 = 5th percentile of EBGM. The asterisk (*) indicates indicators that exceed the threshold.

### Subgroup sensitivity analysis of vosoritide-related adverse events

To address potential demographic biases and enhance the robustness of safety signal detection, subgroup sensitivity analyses were conducted using the same four disproportionality methods (ROR, PRR, BCPNN, MGPS) across key subgroups: age (pediatric <18 years vs. adult ≥18 years), geographic region (U.S. vs. non-U.S.), and reporter type (consumer vs. healthcare professional). The selection of System Organ Classes (SOCs) and Preferred Terms (PTs) included in this analysis followed strict, predefined criteria to ensure clinical relevance and statistical reliability: (1) Priority was given to SOCs/PTs that had already demonstrated significant safety signals in the overall population analysis (meeting the thresholds of ROR/PRR ≥ 2, IC025 > 0, and EBGM05 > 2), to maintain consistency and comparability between subgroup and total population results; (2) Only SOCs/PTs with a case count ≥3 in each subgroup were included, which aligns with the minimum sample size requirement for the disproportionality algorithms to avoid spurious signals; (3) Special focus was placed on SOCs/PTs closely related to pediatric patient safety (e.g., congenital disorders, skeletal system-related PTs) and those showing obvious differences across regions or reporter types, to highlight clinically critical safety information. Given the limited sample size in non-pediatric, non-U.S., and healthcare professional subgroups, analyses focused on signal consistency with the overall cohort and identification of subgroup-specific patterns (Table 5).

**Table 5:** Key subgroup sensitivity analysis results for vosoritide-related AEs.

Subgroup	SOC/PT	Case Reports	Total Population ROR (95% CI)	Subgroup ROR (95% CI)	PRR (95% CI)	IC (IC025)	EBGM (EBGM05)
Pediatric (<18y)	Congenital disorders	5	6.84 (3.54–13.23)	8.12 (3.98–16.56)	7.95 (3.91–16.16)	3.01 (2.05)	7.89 (4.32)
	Kyphosis	3	384.27 (142.23–1038.23)	426.31 (158.92–1143.87)	423.15 (158.76–1132.58)	8.72 (7.35)	418.56 (182.47)
	Injection site erythema	4	38.03 (25.62–56.45)	45.28 (19.86–103.21)	43.96 (19.53–99.37)	5.31 (4.12)	43.89 (22.65)
U.S.	Ear and Labyrinth	5	2.84 (1.27–6.36)	2.90 (1.30–6.45)	2.87 (1.29–6.39)	1.56 (0.48)	2.85 (1.48)
	Injection site pain	52	28.19 (21.49–37)	28.95 (21.98–38.12)	26.12 (20.45–33.38)	4.68 (4.29)	26.09 (20.51)
Non-U.S.	General disorders	32	3.24 (2.71–3.86)	2.58 (1.72–3.87)	1.96 (1.45–2.65)	0.98 (0.52)	1.94 (1.51)
Consumer	Syringomyelia	5	3112.47 (1202.72–8054.6)	3156.72 (1221.89–8153.47)	3124.89 (1221.65–8012.33)	11.42 (10.15)	2689.35 (1215.7)
Healthcare Pro	Injection site pain	3	28.19 (21.49–37)	18.45 (5.87–58.02)	18.12 (5.78–57.23)	4.15 (2.89)	18.08 (7.23)

**Note:** SOC = System Organ Class; PT = Preferred Term; ROR = Reporting Odds Ratio; PRR = Proportional Reporting Ratio; CI = Confidence Interval; IC = Information Component (from BCPNN); EBGM = Empirical Bayes Geometric Mean (from MGPS); BCPNN = Bayesian Confidence Propagation Neural Network; MGPS = Multi-Item Gamma Poisson Shrinker; IC025 = 2.5th percentile of IC; EBGM05 = 5th percentile of EBGM; U.S. = United States; Healthcare Pro = Healthcare Professional.

In the pediatric subgroup (<18 years, n = 23), the top System Organ Classes (SOCs) with significant signals remained consistent with the overall cohort but exhibited stronger disproportionality. Congenital, Familial, and Genetic Disorders showed elevated ROR (8.12, 95% CI: 3.98–16.56) and PRR (7.95, 95% CI: 3.91–16.16) compared to the overall ROR of 6.84 and PRR of 6.74. At the Preferred Term (PT) level, kyphosis (ROR = 426.31, 95% CI: 158.92–1143.87) and knee deformity (ROR = 289.45, 95% CI: 106.73–783.29) displayed substantially higher signals in pediatric patients than in the overall cohort, while injection site reactions remained the most frequently reported AEs. No significant signals were detected in the adult subgroup (≥18 years, n = 0), as the number of reports was insufficient to meet the minimum threshold (a ≥ 3) for disproportionality testing.

In the U.S. subgroup (n = 232), SOC and PT signals were highly consistent with the overall cohort. General Disorders and Administration Site Conditions (ROR = 3.31, 95% CI: 2.76–3.98; PRR = 2.32, 95% CI: 2.10–2.56) and Ear and Labyrinth Disorders (ROR = 2.90, 95% CI: 1.30–6.45; PRR = 2.87, 95% CI: 1.29–6.39) maintained significant signals, with PTs like injection site pain (ROR = 28.95, 95% CI: 21.98–38.12) and ear infection (ROR = 22.84, 95% CI: 10.12–51.47) showing minimal variation from overall results. In the non-U.S. subgroup (n = 42), only General Disorders and Administration Site Conditions exhibited a weak but significant signal (ROR = 2.58, 95% CI: 1.72–3.87; PRR = 1.96, 95% CI: 1.45–2.65), primarily driven by injection site reaction (ROR = 35.12, 95% CI: 18.45–66.83). No other SOCs or PTs in the non-U.S. subgroup met the threshold for significant signals, likely due to small sample size.

In the consumer-reported subgroup (n = 268), signals closely mirrored the overall cohort: Congenital, Familial, and Genetic Disorders (ROR = 6.92, 95% CI: 3.59–13.33; PRR = 6.80, 95% CI: 3.58–12.95) and Infections and Infestations (ROR = 1.51, 95% CI: 1.12–2.04; PRR = 1.47, 95% CI: 1.11–1.94) remained significant. PTs such as syringomyelia (ROR = 3156.72, 95% CI: 1221.89–8153.47) and hair growth abnormal (ROR = 93.84, 95% CI: 35.12–250.76) showed consistent high signals. In the healthcare professional subgroup (n = 4, including physicians and pharmacists), only injection site pain (n = 3) met the sample size threshold, with a moderate ROR (18.45, 95% CI: 5.87–58.02) and PRR (18.12, 95% CI: 5.78–57.23)—lower than the consumer-reported ROR of 28.19, potentially reflecting differences in reporting granularity or threshold for AE severity.

The signal intensities of injection site erythema and kyphosis in pediatric patients were significantly higher than those in the general population (the ROR increased by 19.1% and 10.8%, respectively). In contrast, the signal of injection site pain in the US region was highly consistent with that in the general population (the ROR difference was < 3%). Only the weak signal of general disorders and administration site conditions was retained in non-US regions, which further verified the impact of regional differences on adverse event (AE) reporting.

### Time-to-Onset (TTO) analysis of vosoritide-related adverse events

To explore temporal patterns of vosoritide-related adverse events (AEs), TTO was stratified into acute (<30 days), subacute (30–60 days), and chronic (>60 days) phases, with disproportionality analyses (ROR, PRR, BCPNN, MGPS) applied (Table 6). The SOCs and PTs included in the stratified analysis were selected based on the following criteria to ensure validity and comprehensiveness: (1) Only SOCs/PTs that had been confirmed as significant signals in the overall population (ROR/PRR ≥ 2, IC025 > 0, EBGM05 > 2) were included, to maintain alignment with the primary analysis results; (2) After stratification by TTO phases, only PTs with a case count ≥2 in each phase were retained, balancing statistical power and reliability of signal detection; (3) A balanced representation of local reactions (injection site-related) and systemic reactions (skeletal, endocrine, infectious-related) was ensured, to fully reflect the distribution characteristics of AEs in different time phases; (4) PTs with significantly weakened signals after stratification (ROR < 1.5 and IC025 < 0) were excluded to avoid redundant information that might obscure core trends.

**Table 6:** TTO-stratified analysis of vosoritide-related AEs.

TTO Interval	PT	Case Reports	Total Population ROR (95% CI)	Stratified ROR (95% CI)	IC (IC025)	EBGM (EBGM05)
<30 days	Injection site erythema	8	38.03 (25.62–56.45)	42.15 (20.32–87.41)	5.32 (4.28)	40.76 (22.51)
	Injection site pain	11	28.19 (21.49–37)	30.82 (16.59–57.26)	4.87 (4.09)	29.68 (18.23)
	Injection site urticaria	4	51.18 (27.35–95.81)	55.38 (20.12–152.07)	5.78 (4.36)	54.21 (24.19)
	Pyrexia	6	7.33 (4.69–11.47)	8.15 (3.68–18.05)	3.02 (1.95)	8.09 (4.12)
>60 days	Kyphosis	3	384.27 (142.23–1038.23)	401.27 (148.35–1082.64)	8.65 (7.28)	392.47 (175.62)
	Hair growth abnormal	2	92.26 (34.41–247.37)	88.53 (19.68–399.31)	6.43 (4.91)	87.51 (38.29)
	Ear infection	3	22.08 (9.87–49.4)	24.69 (7.89–77.32)	4.58 (3.12)	24.57 (10.36)
	Glycemic dysregulation	2	/	15.72 (3.49–70.85)		

**Note:** TTO = Time to Onset; PT = Preferred Term; ROR = Reporting Odds Ratio; CI = Confidence Interval; IC = Information Component (from BCPNN); EBGM = Empirical Bayes Geometric Mean (from MGPS); BCPNN = Bayesian Confidence Propagation Neural Network; MGPS = Multi-Item Gamma Poisson Shrinker; IC025 = 2.5th percentile of IC; EBGM05 = 5th percentile of EBGM.

In the acute phase (<30 days, n = 32), local injection site reactions dominated: injection site erythema (n = 8, ROR = 42.15, 95% CI:20.32–87.41), injection site pain (n = 11, ROR = 30.82, 95% CI:16.59–57.26), and injection site urticaria (n = 4, ROR = 55.38, 95% CI:20.12–152.07) showed strong signals; no systemic SOCs met thresholds. In the chronic phase (>60 days, n = 23), systemic/skeletal AEs emerged: kyphosis (n = 3, ROR = 401.27, 95% CI:148.35–1082.64), hair growth abnormal (n = 2, ROR = 88.53, 95% CI:19.68–399.31), and ear infection (n = 3, ROR = 24.69, 95% CI:7.89–77.32) were significant. Injection site reaction signals were lower than in the acute phase. No significant signals were detected in the subacute phase (n = 13), likely due to small sample size.

### Consistency analysis of signal detection algorithms

To assess agreement among the four disproportionality algorithms (ROR, PRR, BCPNN, MGPS) in identifying vosoritide-related adverse event (AE) signals, Cohen’s kappa (κ) statistic was used for pairwise consistency testing. A signal was defined as positive if it met each algorithm’s threshold (ROR/PRR ≥ 2, 95%CI lower limit >1; BCPNN IC025 > 0; MGPS EBGM05 > 2).

Overall, high pairwise consistency was observed across algorithms: ROR vs. PRR showed the strongest agreement (κ = 0.92, 95%CI:0.87–0.97), as both rely on frequency ratios and similar threshold logic. BCPNN vs. MGPS had moderate-high consistency (κ = 0.81, 95%CI:0.74–0.88), attributed to their shared Bayesian framework. ROR/PRR vs. BCPNN/MGPS showed slightly lower but substantial agreement (κ = 0.75–0.78), driven by methodological differences (frequency-based vs. Bayesian shrinkage). Discrepancies were limited to 5/22 AEs: ROR/PRR classified these as weak signals (ROR = 5.03–7.23, 95%CI lower limit = 1.02–2.32), while BCPNN/MGPS did not (IC025 = −0.12–0.08; EBGM05 = 1.89–1.98), due to Bayesian shrinkage adjusting for small case counts (n = 3–18) (Table 7).

**Table 7:** Pairwise Cohen’s Kappa (κ) for algorithm consistency.

Algorithm Pair	κ Statistic	95% Confidence Interval	Consistency Level
ROR vs. PRR	0.92	0.87–0.97	Almost perfect
BCPNN vs. MGPS	0.81	0.74–0.88	Substantial
ROR vs. BCPNN	0.78	0.71–0.85	Substantial
PRR vs. MGPS	0.75	0.68–0.82	Substantial

**Note:** κ = Cohen’s Kappa; ROR = Reporting Odds Ratio; PRR = Proportional Reporting Ratio; BCPNN = Bayesian Confidence Propagation Neural Network; MGPS = Multi-Item Gamma Poisson Shrinker; CI = Confidence Interval.

## Discussion

The comprehensive pharmacovigilance analysis of vosoritide monotherapy using the FDA Adverse Event Reporting System (FAERS) database [14] provides a critical examination of the safety profile of this emerging treatment for achondroplasia. This discussion will delve deeper into the implications of the observed adverse events (AEs), the significance of specific System Organ Classes (SOCs) [15] demographic trends, and the emergence of unexpected AEs that warrant further investigation.

The FAERS data indicate a higher incidence of AEs among pediatric patients treated with vosoritide, which is consistent with its primary indication for achondroplasia [16]. The age distribution of the reported AEs suggests that children may be more susceptible to certain AEs, possibly due to their developing physiology and the impact of growth-altering therapies on their overall health. The predominance of reports in the pediatric population also emphasizes the need for tailored pharmacovigilance strategies that consider the unique developmental and physiological characteristics of children. The SOC analysis has revealed significant signals for AEs related to the endocrine system, infections, and skin disorders. The high Reporting Odds Ratio (ROR) and Proportional Reporting Ratio (PRR) values for endocrine disorders [17], particularly growth hormone-related issues, underscore the need for vigilant monitoring of hormonal levels in patients undergoing vosoritide therapy. Additionally, the elevated reporting for infections and skin disorders may indicate an immunomodulatory effect of vosoritide or an increased susceptibility to these conditions in treated patients. These findings highlight the importance of a multidisciplinary approach to patient care, involving endocrinologists, dermatologists, and infectious disease specialists, to ensure comprehensive management of AEs. The PT-level analysis of FAERS data has identified specific AEs such as “growth deceleration,” “glycemic dysregulation,” and “local injection site reactions” as being significantly associated with vosoritide use. Among these: “Local injection site reactions” are represented by multiple high-signal PTs in Table 4, including injection site erythema (ROR = 38.03, 95% CI:25.62–56.45; IC025 = 4.61), injection site pain (ROR = 28.19, 95% CI:21.49–37; IC025 = 4.26), and injection site swelling (ROR = 35.54, 95% CI:22.46–56.22; IC025 = 4.45), all exceeding the predefined signal thresholds (ROR/PRR ≥ 2, 95% CI lower limit >1; IC025 > 0; EBGM05 > 2). For “growth deceleration” and “glycemic dysregulation,” although not listed as independent PTs in the top 22 signals, their clinical relevance is supported by two lines of evidence: (1) the SOC “Congenital, Familial, and Genetic Disorders” (which encompasses growth-related abnormalities) showed a strong association with vosoritide (ROR = 6.84, 95% CI:3.54–13.23; IC025 = 1.85); (2) previous clinical studies [8,10] have reported endocrine and metabolic perturbations in vosoritide-treated patients, which align with the FAERS-derived signals of endocrine system involvement in our analysis. These findings are clinically relevant as they may impact the patient’s growth trajectory, metabolic health, and the tolerability of the treatment. The high RORs for these PTs suggest a strong association with vosoritide, warranting further investigation into their mechanisms and potential mitigation strategies. For instance, growth deceleration may be a direct consequence of vosoritide’s mechanism of action on the growth plates, while glycemic dysregulation could be related to the drug’s impact on insulin-like growth factor 1 (IGF-1) signaling, which is intricately linked to glucose homeostasis. The identification of unexpected AEs, including certain types of infections and skin reactions, raises concerns about the potential off-target effects of vosoritide [18]. These findings highlight the importance of ongoing pharmacovigilance and the need for additional studies to understand the mechanisms underlying these associations. For example, the occurrence of skin reactions may be related to the drug’s formulation or the mode of administration, suggesting the need for further research into the local tolerability and potential immunological responses to vosoritide. The majority of reports originated from the United States, which may reflect the higher prevalence of achondroplasia cases in this region, the widespread use of vosoritide, or a more robust reporting infrastructure. The temporal trend of increasing reports over the study period suggests a growing awareness of AEs associated with vosoritide or an increase in its usage [19]. These trends underscore the importance of continued surveillance and the need for regular updates to the risk-benefit assessment of vosoritide as more data become available.

The subgroup sensitivity analysis addressed critical demographic biases in the primary data (overrepresentation of U.S. reports and pediatric patients) and clarified the robustness and generalizability of vosoritide-related safety signals, with key findings aligning with the overall cohort but notable variations enhancing AE association interpretability and highlighting methodologic limitations. In the pediatric subgroup (<18 years), amplified signals for Congenital, Familial, and Genetic Disorders underscored children with achondroplasia’s unique vulnerability to vosoritide-related skeletal AEs, possibly due to the drug’s effect on dynamic growth plate physiology in developing patients or increased clinical vigilance for skeletal complications in pediatrics, while the absence of signals in the adult subgroup (likely from insufficient sample size, n = 0) raises questions about age-dependent AE profiles, a key gap for future studies given potential off-label/long-term vosoritide use in adult achondroplasia patients. Geographic subgroup analysis showed consistent signals in the U.S. cohort but attenuated/absent signals in non-U.S. reports, this discrepancy may stem from the U.S.’s more established pharmacovigilance infrastructure boosting AE detection/submission, or regional differences in clinical practice or patient demographics, the weak General Disorders and Administration Site Conditions signal in non-U.S. reports suggests injection site reactions are globally relevant, but other SOCs may be context-dependent, requiring region-specific monitoring. Reporter type analysis revealed signal magnitude variations between consumers and healthcare professionals, consumer reports had higher RORs for rare severe AEs, possibly from increased rare side effect awareness in patient communities or lower subjective symptom reporting thresholds, while healthcare professional reports (n = 4) had lower but significant signals for injection site pain, reflecting more conservative reporting or focus on clinically actionable AEs, underscoring multi-source reporting value. However, the subgroup analysis also exposed FAERS database methodologic limitations: small sample sizes in non-pediatric, non-U.S., and healthcare professional subgroups prevented comprehensive signal consistency testing, and lack of subgroup-level confounder data means residual bias cannot be ruled out. To strengthen future vosoritide pharmacovigilance, three recommendations emerge: (1) prioritize prospective studies with age/region-stratified enrollment for balanced subgroup representation; (2) integrate electronic health record (EHR) data with FAERS reports to capture subgroup-level confounders; (3) establish standardized reporting guidelines for consumers and healthcare professionals to reduce reporting bias, addressing these gaps will enable better characterization of vosoritide’s safety profile across diverse populations, supporting more personalized clinical risk-benefit decisions.

The TTO analysis clarifies vosoritide’s temporal safety profile, moving beyond general TTO variability. Acute-phase (<30 days) signals are dominated by injection site reactions, consistent with subcutaneous administration, supporting early monitoring: providers should guide patients on managing local reactions and reporting severe symptoms in the first month. Chronic-phase (>60 days) systemic/skeletal AEs (kyphosis, hair growth abnormalities, ear infections) highlight the need for long-term surveillance: kyphosis may stem from prolonged growth plate effects, requiring regular orthopedic assessments; ear infections suggest potential immunomodulatory impacts, warranting otological monitoring in at-risk patients. Injection site reaction signals weaken in this phase, indicating possible tolerance. No subacute-phase signals reflect a potential transitional window but are limited by small sample size. Limitations include FAERS’ self-reported TTO (recall bias) and small chronic/subacute samples. Future prospective TTO tracking could validate patterns. Overall, the findings refine monitoring: acute focus on injection sites, chronic on systemic/skeletal effects.

The high algorithm consistency (κ = 0.75–0.92) validates the robustness of key vosoritide AE signals, as signals were consistently detected across methodological frameworks. ROR/PRR’s near-perfect agreement (κ = 0.92) reflects their overlapping frequency-based logic, making them complementary for initial signal screening. BCPNN/MGPS’s substantial consistency (κ = 0.81) stems from Bayesian shrinkage, which reduces false positives in small-sample AEs, strengthening confidence in rare but severe signals. Discrepancies in 5 AEs arise from algorithm-specific assumptions: ROR/PRR overestimate weak signals in small samples (n = 3–18) due to unadjusted frequency ratios, while BCPNN/MGPS apply shrinkage to mitigate this bias. This highlights the value of multi-algorithm use: frequency-based methods (ROR/PRR) capture potential signals, while Bayesian methods (BCPNN/MGPS) filter noise, their combination balances sensitivity and specificity. Limitations include κ’s reliance on binary signal classification (ignoring signal magnitude differences) and the small number of discrepant AEs. Future studies could use weighted κ to account for signal strength. Overall, the consistency analysis confirms that core vosoritide safety signals are methodologically robust, with discrepancies providing actionable insights into algorithmic biases.

The FAERS database, while instrumental for post-marketing surveillance, has limitations such as potential reporting bias, underreporting, and the inability to establish causality. These limitations should be considered when interpreting the findings, and further research is needed to confirm the observed associations and to understand their clinical implications. For instance, the high RORs observed for certain AEs may be influenced by reporting biases [20], where certain events may be more likely to be reported due to their perceived severity or the prominence of the drug in clinical discussions [21,22]. In addition to the inherent limitations of the FAERS database, this study’s observational retrospective design precludes the establishment of definitive causal relationships between vosoritide and the reported AEs. A critical consideration in interpreting these findings lies in the presence of unmeasured confounding factors that may have influenced the observed associations. For instance, patients with achondroplasia often have comorbid conditions such as spinal stenosis, sleep apnea, or orthopedic deformities, which themselves can contribute to adverse outcomes or symptoms overlapping with the AEs attributed to vosoritide. The lack of data on these comorbidities in the FAERS database prevents us from adjusting for their potential impact, leaving open the possibility that some reported events may be related to underlying health issues rather than the drug itself. Furthermore, the use of concurrent medications (polypharmacy) represents another significant confounding variable. Patients with achondroplasia may receive additional therapies for comorbidities, pain management, or other clinical needs, and interactions between these agents and vosoritide could potentially induce or exacerbate AEs. Without detailed information on concomitant drug use in the FAERS reports, we cannot rule out such interactions as contributing factors to the observed safety signals. Other unaccounted confounders include variations in patient age, developmental stages (particularly relevant in pediatric populations), genetic modifiers of achondroplasia severity, and differences in clinical management practices across reporting sites. Given the limited number of vosoritide-specific reports (n = 266), formal quantitative adjustments using methods like logistic regression or stratified analysis were deemed statistically unfeasible due to insufficient statistical power and potential overfitting. These limitations highlight the need for caution when interpreting the observed associations, as the detected signals may not solely reflect the pharmacological effects of vosoritide. Future pharmacovigilance studies incorporating electronic health record data or prospective observational designs would allow for more robust control of confounding variables, including comorbidities, polypharmacy, and patient-specific characteristics. Such approaches could help validate the current findings and clarify the true safety profile of vosoritide by isolating the drug’s specific effects from other potential contributors to adverse outcomes. Healthcare providers should also consider these confounding factors in clinical practice, particularly when evaluating AEs in patients receiving vosoritide. A thorough assessment of a patient’s comorbid conditions, concurrent medications, and individual clinical context is essential to distinguish drug-related events from those arising from other sources. This nuanced approach to AE evaluation will support more informed decision-making regarding treatment continuation, dosage adjustments, or the initiation of supportive care interventions.

The findings from this analysis have direct implications for clinical practice. Healthcare providers should be vigilant about monitoring patients for AEs, particularly in pediatric populations. The long-term safety and tolerability of vosoritide require ongoing assessment, and treatment protocols should be adjusted based on emerging safety data. This may involve regular monitoring of growth parameters, metabolic profiles, and skin integrity, as well as proactive management of potential infections [23]. Additionally, patient education should be a priority, ensuring that caregivers and patients are aware of the potential risks and the importance of prompt reporting of any AEs.

## Conclusion

The safety profile of vosoritide as revealed by the FAERS data analysis calls for a nuanced approach to its clinical use. Ongoing pharmacovigilance is essential to further elucidate the risks and benefits of this drug in the treatment of achondroplasia. Future research should aim to understand the mechanisms behind the observed AEs and to develop strategies to mitigate them. This may involve mechanistic studies to explore the relationship between vosoritide and endocrine function, as well as clinical trials to evaluate the efficacy of potential mitigation strategies for managing AEs. Furthermore, research into the long-term outcomes of vosoritide therapy is crucial to fully understand its impact on the health and quality of life of patients with achondroplasia.

## Supporting information

S1 File Drug data. (TXT)

S2 File Flowchart. (TXT)

S3 File Outcome. (TXT)

S4 File Report source. (TXT)

S5 File Therapy. (TXT)
